# IOM Issues Report on Breast Cancer and the Environment

**DOI:** 10.1289/ehp.120-a60a

**Published:** 2012-02-01

**Authors:** Charles W. Schmidt

**Affiliations:** Charles W. Schmidt, MS, an award-winning science writer from Portland, ME, has written for *Discover Magazine*, *Science*, and *Nature Medicine*.

The Institute of Medicine (IOM) recently set out to review the current evidence on links between breast cancer and the environment. Their conclusions, published in a report issued 7 December 2011,^1^ point to a range of actions women can take to reduce their risk: maintain a healthy weight, limit alcohol use, don’t smoke, forego certain forms of postmenopausal hormone therapy, and avoid excessive medical imaging when possible. Conclusive evidence links each of these factors to breast cancer, while the evidence supporting other factors—notably exposure to industrial and workplace chemicals—remains less certain, according to the report’s authors.

Commissioned by Susan G. Komen for the Cure®, a Dallas, Texas–based cancer research advocacy group, the report was prepared by a 15-member panel from academia and community health centers. The panel defined “environment” as any factor that isn’t inherited through DNA and relied on evidence compiled by the International Agency for Research on Cancer and the World Cancer Research Fund International, in addition to findings from the scientific literature.

Breast cancer risk factors were divided into 3 categories—established, possible, and biologically plausible—on the basis of the strength of the data, according to panelist Robert Hiatt, a professor of epidemiology and biostatistics at the University of California, San Francisco. “Established” risk factors were supported by strong human epidemiologic data, in addition to positive results from animal and mechanistic studies. Risk factors were assigned a “possible” status if the available human data were in conflict and a “biologically plausible” status if they were supported solely by animal and mechanistic studies.

Remarkably, only a few established risk factors were identified, among them combined hormone therapy with estrogen and progestin, exposure to ionizing radiation (such as that delivered by computed tomographic scans), excess weight in postmenopausal women, and excessive alcohol use. Possible risk factors include nighttime shift work and exposure to secondhand smoke, benzene, ethylene oxide, and 1,3-butadiene. The biologically plausible category is populated mainly by industrial chemicals, including metals, pesticides, and the plastics constituent bisphenol A, which is ubiquitous in consumer products, the environment, and people, and therefore exceedingly challenging to study in controlled epidemiologic studies, according to Hiatt.

By emphasizing lifestyle changes in prevention, the IOM distinguished itself from the President’s Cancer Panel (PCP), which in spring 2010 released a headline-grabbing annual report^2^ stressing that chemical exposures have a “grossly underestimated” impact on cancer risk.^3^ The first of the PCP’s annual reports to focus specifically on the environment’s role in cancer, it called explicitly for tightening regulations on chemical exposure, but the IOM avoids any similar recommendation. The new report does state that laboratory data linking chemicals to human cancer hazards “may well warrant consideration of actions by regulatory agencies that are aimed at reducing future population-based exposures.”

But Hiatt says the IOM panel was charged with reviewing the current evidence, not recommending regulatory policy. In most cases, he says, more human data are needed to bolster cause–effect relationships between chemicals and breast cancer. “We usually couldn’t find solid human evidence of effect,” he says. “Either the data wasn’t there or it was conflicting, and we had to make the call based on what we know in 2011.”

The chief recommendation of the IOM is that researchers adopt a “life course” approach to studying breast cancer and the environment, with more emphasis on early-life human exposure. “One reason that we might be missing the boat on the human data is that we’re looking at adult women, while carcinogenic effects may result from exposures that happen while the breast is still developing,” Hiatt says. “We know from animal experiments that this is a window of vulnerability to chemical insults.”

**Figure:**
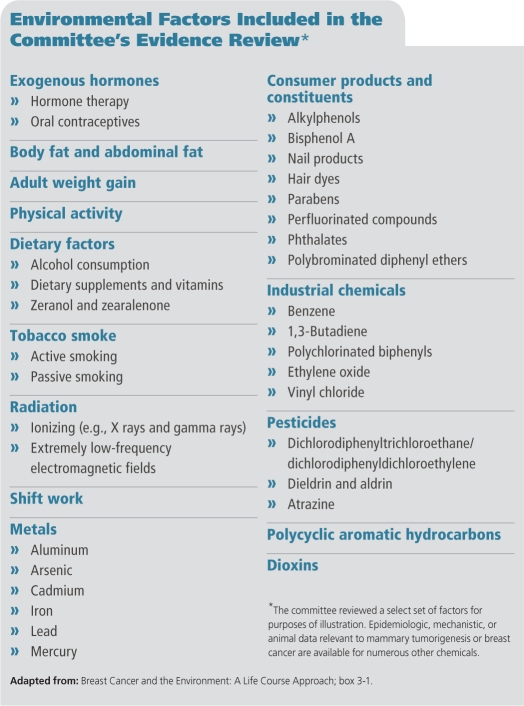
Environmental Factors Included in the Committee’s Evidence Review

Along those lines, Hiatt adds, the National Institute of Environmental Health Sciences and the National Cancer Institute are collaborating on the Breast Cancer and the Environment Research Program, which recruits subjects starting at the age of 6 years. The two institutes also collaborate on the Interagency Breast Cancer and Environmental Research Coordinating Committee, a congressionally mandated body currently preparing a comprehensive report on federal research on the environmental and genomic factors related to breast cancer. This report is expected in mid-2012.

Michael Thun, vice president emeritus of surveillance and epidemiology research at the American Cancer Society, lauds the IOM report for systematically reviewing the available evidence. “It fills an important gap,” he says. “I agree with its position that we have issues of concern as research needs, but they’re in a different category than well-established risk factors.”

Adds Diana Rowden, vice president for survivorship and outcomes at Susan G. Komen for the Cure, “The report shows that women can reduce their breast cancer risk with actionable items undertaken now. And it challenges us to think more about how we explain cancer risk to the general population—about how different factors have their own unique influence on risk. That’s an important takeaway point.”
